# Leaf Curl Epidemic Risk in Chilli as a Consequence of Vector Migration Rate and Contact Rate Dynamics: A Critical Guide to Management

**DOI:** 10.3390/v15040854

**Published:** 2023-03-27

**Authors:** Buddhadeb Roy, Emmadi Venu, Sathiyaseelan Kumar, Shailja Dubey, Dilip Lakshman, Bikash Mandal, Parimal Sinha

**Affiliations:** 1 Division of Plant Pathology, ICAR-Indian Agricultural Research Institute, New Delhi 110012, India; 2 Sustainable Agricultural Systems Laboratory, USDA-ARS, Beltsville, MD 20705, USA

**Keywords:** chilli leaf curl virus, ChiLCV, migrant viruliferous vectors, contact rate, *Bemisia tabaci*, vector interception, epidemic arrest, begomovirus

## Abstract

Chilli is an important commercial crop grown in tropical and subtropical climates. The whitefly-transmitted chilli leaf curl virus (ChiLCV) is a serious threat to chilli cultivation. Vector migration rate and host–vector contact rate, the major drivers involved in the epidemic process, have been pinpointed to link management. The complete interception of migrant vectors immediately after transplantation has been noted to increase the survival time (to remain infection free) of the plants (80%) and thereby delay the epidemic process. The survival time under interception (30 days) has been noted to be nine weeks (*p* < 0.05), as compared to five weeks, which received a shorter period of interception (14–21 days). Non-significant differences in hazard ratios between 21- and 30-day interceptions helped optimize the cover period to 26 days. Vector feeding rate, estimated as a component of contact rate, is noted to increase until the sixth week with host density and decline subsequently due to plant succulence factor. Correspondence between the peak time of virus transmission or inoculation rate (at 8 weeks) and contact rate (at 6 weeks) suggests that host succulence is of critical importance in host–vector interactions. Infection proportion estimates in inoculated plants at different leaf stages have supported the view that virus transmission potential with plant age decreases, presumably due to modification in contact rate. The hypothesis that migrant vectors and contact rate dynamics are the primary drivers of the epidemic has been proved and translated into rules to guide management strategies.

## 1. Introduction

Chilli is an important commercial crop grown in India, Indonesia, China, Japan, Indonesia, Spain, and Turkey. Leaf curl disease in chilli is one of the most economically significant diseases. It is known to be caused by several begomoviruses, of which chilli leaf curl virus (ChiLCV) is the most predominant in India [1]. The virus is efficiently spread by whitefly (*Bemisia tabaci*), which is abundant year-round in tropical and subtropical climates containing a wide variety of reservoir hosts [2]. In addition, semi-natural habitats, border vegetations (legumes and cucurbits on iron hurdles), and weeds also serve as a means for survival [3]. Recently, whitefly-transmitted viruses have become an increasingly influential group of harmful pathogens of vegetables throughout the tropical and subtropical world [4,5,6]. Begomoviruses (family Geminiviridae) are the most destructive plant viruses that cause diseases, such as leaf curl, mosaic, yellow mosaic, and yellow vein mosaic, in numerous crop plants [7]. In chilli, infected plants show upward leaf-curling, boat-shaped downward curling, distortion, vein yellowing, or more generalized leaf yellowing and/or stunted growth with a bushy appearance [8]. Therefore, symptoms associated with leaf curl appear to be complex and compounded by simultaneous attacks by thrips and mites, along with the viruses they carry from various hosts such as legumes, solanaceous, and cucurbits [9,10]. In terms of yield loss, leaf curl has been the most damaging across all cropping regions [1]. In severe cases, up to 100% losses of marketable fruits have been reported [9,10]. Plant growth and development are severely impaired due to the interruption of major biosynthetic processes where both viral infection and direct vector feeding play significant roles in inducing leaf deformation and abnormal growth [5]. Furthermore, the early growth or succulent stage is highly vulnerable to sucking pests in chilli (whiteflies, thrips, aphids, and mites) and the viruses they transmit [11].

The management of leaf curl diseases remains a pressing issue. Barrier or mixed crops, repellent, and pesticide applications aimed at checking vectors remain ineffective due to a lack of clear perception of dynamic tripartite (i.e., ChiLCV–whitefly–chilli plant) interactions in the epidemic process. Furthermore, precise information on the etiological association with complex symptoms, epidemiological insights, and phenological affiliation with vulnerability is insufficient and yet to be resolved cohesively. An in-depth epidemiological understanding of the tripartite interactions to predict and manage their consequences is necessary to evolve a management strategy.

Leaf curl epidemic in chilli is the result of tripartite interactions between host–vector–virus, which is further complicated by simultaneous attacks of thrips and mites [11,12,13]. The progress of tripartite interactions over time is influenced by several factors associated with the host and vectors and modified by temperature [11]. Host susceptibility, age, cropping intensity, spatial distribution, and the potency of infected hosts are the crucial factors that modify the interaction process [14,15,16]. The contact rate between the viruliferous vector and the growing host is crucial in epidemic development [17,18]. Therefore, the number of viruliferous vectors, their mobility, spatial distribution, and host behaviour immensely affect the contact rate [11]. Changes in host growth and vector population over time will likely alter the contact rate, resulting in variation in the virus transmission rate. Therefore, periods of high contact appear to be critical points of intervention [17]. Precise intervention to lower contact rates that reduces the proportion of infectious hosts may provide clues for the development of management guides [19]. Assessing contact rate dynamics and its link with epidemic development requires a framework model incorporating tripartite interactions [20]. Population dynamical models to explain tripartite interactions for vector-borne virus diseases have been used for testing intervention strategies [21,22]. The prediction of leaf curl dynamics in chilli has indicated that the immigration rate of vector population is a sensitive parameter at equilibrium [11]. However, it is critical to understand the underlying mechanisms in the epidemic process. The integration of tripartite interactions in agroecological perspectives and various factors affecting migration rate and host–vector contact rates is critical to understand the mechanisms that ultimately drive the epidemic. For plant viruses, the contact rate between the host and vector has not been studied in great detail. Management practices solely based on vector density, therefore, have not been successful. For mosquito-borne virus diseases, it is well established that the contact rate between the host and vector plays an important role in driving the epidemic [20,23,24]. Host–vector contact dynamics play a significant role in driving the epidemic that integrates many factors eventually leading to virus transmission [20]. Therefore, it is vital to understand the vector feeding behaviour or host preference, and their application in disease control [14]. A conceptual framework assessing transmission risk is necessary to identify the relative importance of extrinsic and intrinsic drivers of risk to design and implement strategic control efforts [25,26,27,28].

In this paper, a prognostic evaluation of the driving parameters—particularly migration rate and contact rate dynamics—shows that they exert an implicitly profound effect on the epidemic process. The impact of a migrant viruliferous vector population is simulated in terms of infectious host and viruliferous vector population to implicate the importance of vector interception from the beginning of the crop growth. The prediction of the peak period of host–vector contact based on the host density and growth stage preference is shown to prove that the interception of migrant carrier vectors at the early growth stage is an effective way to reduce contact rate and thereby check leaf curl epidemic. Furthermore, the early growth phase of plants and its conduciveness for both vector feeding and virus transmission, and their effect on plant growth rate, provides useful guidance for the development of an effective management policy.

## 2. Materials and Methods

### 2.1. Prognostication of Major Driver in Chilli Leaf Curl Epidemic in Population Dynamic Modelling Framework

Conceptually, to explore and analyse the most critical parameter in the leaf curl epidemic process, tripartite interactions in the chilli–whitefly–virus transmission system were considered. Leaf curl epidemic as the progress of tripartite interactions was described through the population dynamic model set up [29,30,31,32,33,34]. Previously, leaf curl dynamics, explained and parameterized using the following set of Equation (1) to (4), were implemented for ascertaining the definite role of the major drivers of the epidemic [11]:

(1) *dS/dt = −aVS*

(2) *dI/dt = aVS*

(3) *dX/dt = cX + cV − bXI/(K_x_ + X) − uV + i_x_ − e_x_*

(4) *dV/dt = bXI/(K_x_ +X) + i_v_ − e_v_ − uV = b(1 − V)I/(K_x_ +1 − V) + i_v_ − e_v_ − uV*

Here, virus transmission between host and vector is dependent on parameters *a* (transmission coefficient or inoculation rate) and *b* (acquisition rate). Parameter *a* determines the transfer rate of the healthy host (*S*) into the infectious category (*I*) and is dependent on the number of healthy hosts (*S*) and viruliferous vector population (*V*). Parameter *b* determines the transfer rate of the healthy vector (*X*), which is dependent on the number of healthy vectors (*X*) and the number of available infectious hosts (*I*). Other parameters included in the model are the rate of immigration and emigration of viruliferous (i_v_ and e_v,_ respectively) and healthy (*i_x_* and *e_x,_* respectively) vectors, *c* and *u* birth and death rate, respectively (for both healthy and viruliferous), and Michaelis–Menten constant (*K_x_*).

**Figure 1:**
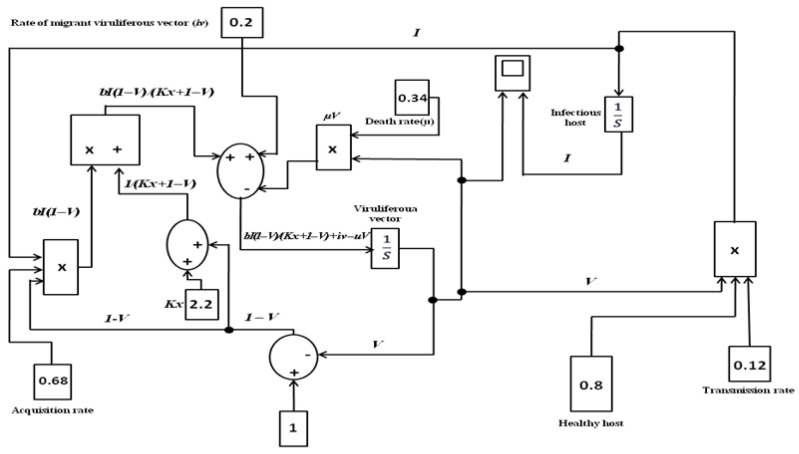
Simulink model for the prediction of leaf curl epidemic explained through the tripartite interactions of host–vector–virus transmission process in chilli.

Hypothetically, to mimic the effect of the migrant viruliferous vector population, three levels of immigration rate were evaluated to observe the changes in leaf curl epidemic in terms of infectious host and viruliferous vector population. Models explaining tripartite interactions were implemented in the Simulink environment (MATLAB 2021a, Mathworks, Natticks, MT, USA) and displayed in Figure 1. The model was also implemented and uploaded github repositores (https://github.com/9873018656, accessed on 30 March 2022). Parameters *a*, *b*, *i_v_*, and *e_v_*, *c*, *u*, and *K_x_* estimated and adjusted by Roy et al. (2021a) [11], were used for the purpose of simulation (Supplementary Table S1). A sensitivity analysis was performed to determine the robustness of the model assessment by examining the extent to which the epidemic is affected by changes in values of the parameter, particularly the rate of the migrant viruliferous vector population. The rate of change in the infectious host population (*I*) was assessed by calculating the derivative (*dI/d**i_v_***), i.e., the rate of change in *I* in terms of the change in the number of migrant viruliferous vectors; otherwise, the rate of immigration of viruliferous vector ***i_v_***.

### 2.2. Field Implementation to Assess the Effect of Complete Interception of Migrant Viruliferous Vector Population on Leaf Curl Epidemic

#### 2.2.1. Experimental Trial for Interception of Migrant Vectors

The interception experiment was conducted in the institute’s experimental site, New Delhi (28.6331° N, 77.1525° E, 219.7 m), from July–December in 2020 and 2021. The site was surrounded by a semi-natural perennial habitat on one side where whitefly eggs, nymphs, as well as adult populations were noted. Two sides had year-round vegetable crops (leguminous, solanaceous, and cucurbits) and another side had a fence with beans and cucurbits. The temperature profile in the site was favourable for whitefly growth and development throughout the year, except for the severe winter period (December–January–mid-February). Plots of size 5 m × 4 m were prepared after the basal application of 10 q/ha of organic manure and NPK (30:30:20). Seedlings of the susceptible genotype (HPH 1041, Maharashtra, India, Syngenta) were raised in a greenhouse chamber to avoid vector and virus infection. Thirty-five-day-old seedlings were transplanted row to row and plant to plant at 45 cm. To check for weeds, black colour polythene mulch was added immediately after transplantation.

For vector interception, a white plant cover with 17 GSM (size of 2 m wide and 400 m in length, Iris Polymers Industries Pvt Ltd., Pune, India) was used to shield the seedlings immediately after transplantation. In addition, a polyethylene stick (70 cm) was used to maintain internal space for plant growth. Three plots, each with 80 plants, were covered immediately after transplanting to ensure complete interception of the migrant vectors; they were removed at 14 (cover 1), 21 (cover 2), and 30 (cover 3) days. To compare the vector interception effect, control plots (without any intervention in vector control) and insecticidal spray plots were maintained with a similar number of plants. For the plot with insecticidal treatment, four sprays with 0.1% imidacloprid (Shri Ram Agro Chemicals, Hapur, UP, India) were applied at weekly intervals.

#### 2.2.2. Survival Time (Plants Remain Uninfected by Virus) Analysis for Vector Interception

For the survival time analysis, 50 plants from each treatment plot (three levels of plant covers, control, and spray) were marked with colour tags. Every week, one top leaf from each of the 50 plants was checked through a PCR test for ChiLCV detection (Supplementary Table S2). Weekly leaf sampling for PCR tests was carried out from the very first week of transplantation and continued until the 15th week. For covered plots, leaf samples were collected after the 14-, 21-, and 30-day cover period was over.

To assess the effect of complete interception (plant cover) on the epidemic, the survival time S(t) for the plants remaining infection free was estimated based on a Kaplan–Meier curve (1956) using software *SPSS 20.0*. Since observation (PCR test) was continued only until the 15th week, the data were in the right censored type. The plants showing PCR positive were marked as an event that had occurred. The survival function S(t) described the probability of a plant remaining uninfected longer than t, and this was defined as S(t) = Pr (T > t), where T is the time until the leaf samples showed PCR positive for ChiLCV. Survival time S(t) was estimated using the equation:

$$S\left(t\right)=\prod_{j=1}^{k}\left(\frac{n_{j}-d_{j}}{n_{j}}\right)$$

$n_{j}$ = number of individual healthy leaves just before time t_j_; $d_{j}$ = number of individual leaves PCR positive for the virus at t_j_ for t_k_ < t < t_(k + 1)_ where k = 1,2,…,r.

A log-rank test was performed to assess whether the Kaplan–Meier survival curves from the subpopulations of various plant cover periods differ significantly from each other. To explain the relationship between survival time and different interception periods, the Cox proportional hazard model was used [35]. The Cox model was expressed by the hazard function denoted by h(t) to interpret as the risk of becoming infected at time t (week).

h(t) = h_0_(t) × exp(βX)

where t represents the survival time; h(t) is the hazard function determined by a single covariate (X = effect of interception); β is the coefficient as a measure of the impact of the effect size of covariate X; h_0_(t) is the baseline hazard and corresponds to the value of the hazard if X equal to zero. The quantity exp (β) is called the hazard ratio (HR); HR > 1 indicates an increase in hazard with time; <1 decrease in hazard and =1 no effect.

### 2.3. Estimation of Crop Growth Parameters and Fresh Fruit Yield under Interception

The relative growth rate was estimated to assess the growth performance of the plants under different levels of interception. For the assessment of relative crop growth rate, plant height was estimated to compare the effects of different interception treatments. Plant height for 10 plants from each treatment (plant cover periods) was measured and expressed as a weekly mean. The relative growth rate (r) for plant height (H) or the rate of gaining new height per unit of existing height was estimated by fitting the model:

r = (1/H) × (dH/dt).

The number of fruits and fresh fruit weight per plant in each interception treatment were estimated and expressed as yield per ha, assuming a standard fruit yield of 20 t/ha [36,37]. Relative yield gain was estimated as fresh fruit yield divided by standard yield, and the increase in yield was scaled in relation to the yield received in the control treatment (without any intervention).

### 2.4. Leaf Curl Incidence in Terms of Characteristic Leaf Curl Symptom Components under Natural Field Conditions and Interception Covers

Leaf curl incidence in terms of typical symptoms was observed in the institute’s experimental site from July to December in 2021. For leaf curl incidence assessment based on symptoms, one top leaf from 200 randomly selected plants was collected weekly (from a field having 350 plants). The leaves were brought into the laboratory, shuffled, and, based on visual symptoms, the proportion of leaf samples for individual symptoms such as mottling/chlorosis, curling/puckering/crinkling, and boat shape were estimated. The severity of leaf symptoms was graded as 0—apparently healthy without any symptoms; 1—the initiation of either curling and/or puckering; 2—distinct mottling, curling, and puckering (both upward and downward); 3—a severe level of mottling, curling, puckering, and crinkling; and 4—a reduction in leaf lamina size and a bushy appearance. Percent leaf curl index (PLCI) was calculated by the formula [38,39];

PCLI = (sum of all grades × 100)/(number of leaves observed × maximum grade)

Similarly, PCLI was also measured in the plants which were under interception covers (14, 21, and 30 days) performed during 2021. For the optimization of cover time, polynomials for PCLI (without and with interception covers) were used to estimate the common intersection between them.

### 2.5. Eventual Assessment of Leaf Curl Incidence under Enhanced Protection in the Interception

A follow-up field experiment was redesigned for interception time with enhanced protection against mites. For mite prevention, seedlings were raised after seed treatment (imidacloprid 5 g/Kg seed) and transplanted after 40 days. Immediately after transplantation, a similar plant cover (Iris Polymers Industries Pvt Ltd.) was used and removed after 26 days. For weed management, black plastic mulch (polythene sheet) was added at the time of transplantation. For a comparison of the interception effect on leaf curl epidemic control plots (without any intervention on vector control), one top leaf from 50 randomly selected plants was collected at the 9th week of transplantation for the evaluation of symptom components. For the control group, the top 60 leaves were randomly collected from the field containing 286 plants. The leaves were brought into the laboratory, shuffled, and visually scored for symptoms of mottling/chlorosis, curling/puckering/crinkling, and boat shape; PCLI was estimated using the formula described, and a PCR test was performed (Supplementary Table S2).

### 2.6. Prediction of Feeding Rate and Approximation of Contact Rate Dynamics Based on Host Density (Leaf Number and Leaf Area) and Succulence Level

For an approximation of contact rate, a mechanistic model explained for mosquito–virus transmission was adopted which estimates vector feeding or predation rate based on host density [20,25]. Contact rate is explicitly defined in the transfer or inoculation rate (*a*) and denoted as the force of infection in Equations (1) and (2):

d*I*/dt = *aVS* = *aV*(1 − *I*)

*a* = *b_vh_* × (*I_v_*/*N_h_*) × *v_h_*;

where *b_vh_* = per capita contact rate that a vector experiences (feeding or predation rate) dependent on host density. It is initially low and increases gradually; *I_v_*/*N_h_* =proportion of bites that are from a viruliferous vector (for well-mixed population *I_v_*/*N_h_*) dependent on the number of viruliferous vectors; and *v_h_* = proportion of bites that results in host infection. Contact rate as a component of feeding rate was estimated based on a mechanistic model derived from the ecological theory called functional response [25,26,27]:

(5) y(*x*) = *A*× *x*/(1 + *A*× *Th* × *x*)

Here, the feeding or predation rate, *y(x)*, is the vector’s per capita feeding rate; *A* is the vector’s search rate (also called discovery rate), and *x* is host density (number of hosts per unit area). The ‘handling time’ per host, *Th*, includes the average time a vector spends interacting with the host, biting the host, and sucking after biting, which is equivalent to inoculation access and feeding period [19,27]. The approach is a compromise between density-dependent and frequency-dependent transmission [25].

#### 2.6.1. Estimation of Host Density (Number of Leaves and Area) and Succulence Level

The susceptible genotype (cv HPH-1041) was raised in a greenhouse chamber to avoid virus infection and vector. Seeds were sown in rectangular pots (80 × 40 cm) with soil media (3/4 garden soil and ¼ organic manure with optimum NPK level). One-month-old seedlings were transplanted in 30 rectangular pots (80 × 40 cm) to maintain about 130–140 plants for observation. The number of leaves was counted from the first week of transplantation, and the weekly mean from 10 plants was estimated. For the estimation of leaf area, the total leaf area of 4–6 plants were measured from RGB images (JPEG) captured through a scanner (dpi 250). Leaf area was measured precisely using image analysis software Assess 2.0 (APS, Paul, MN, USA). To assess the dynamics of leaf number and leaf area, logistic model (Y) was fitted to find the inflection point.

(6) Y(t) = c/(1 + b exp(−a × t))

where Y indicates the proportion of leaf number and leaf area (mm^2^); model parameters c = maximum carrying capacity for leaf number/plant or area/plant; a= apparent rate of growth in leaf number; b = c/X (0)–1. Parameter estimates for leaf number and leaf area were determined using SPSS 16.0.

Leaf succulence (LS) was estimated by measuring leaf fresh weight (FLW) and then dry, and (DW) and LS was expressed [40,41]:

(7) LS = (FLW − DW)/area

Estimates of the feeding or predation rate y(x) or the vector’s per capita feeding rate was a straight line, which is unrealistic in natural situations. Therefore, y(x) values were corrected by multiplying with the succulence level and expressed as:

(8) y (*x*) = *A* × *x*/(1 + *A* × *Th* × *x*) × *LS*

#### 2.6.2. Plant Age and Virus Transmission Potential

The relationship between plant age and infection proportion over a period of time (from the time of inoculation up to the 10th week from transplantation) was assessed to indicate transmission potential. For the assessment of plant age in relation to proportion of infection, a semi-controlled experiment was conducted on the susceptible chilli cultivar (HPH-1041). Seedlings of six successive ages, indicated by the number of leaves, were raised by sowing at weekly intervals. For virus inoculation, four sets of seedlings of different ages (2, 4, 8 and 12leaves) were transplanted in polyethylene pots (dia. 12 cm) and kept under insect-proof cages. Three plants in each set were inoculated with 10 viruliferous whiteflies and maintained at 25 °C to ensure maximum inoculation by the vector [42]. Whiteflies were killed after 48 h by spraying insecticide (@1 mL/L of Confidor, Bayer). The weekly proportion of infection, appropriated as transmission potential in each category, was estimated by testing the leaf samples for ChiLCV. For specific PCR-detection, three top leaves from each of three plants were collected, and the proportion of ChiLCV infection was estimated following the scheme given in Supplementary Table S2. To calculate the proportion of infected leaf (P), the maximum likelihood procedure was used [42,43,44,45]:

P = 1 − ((n − X)/n)^1/m^

where n = number of groups made for leaf samples; m = number of leaves in each group; X = number of groups tested as PCR-positive.

## 3. Results

### 3.1. Prediction of Leaf Curl Epidemic in Response to Migration Rate Parameter

**Figure 2:**
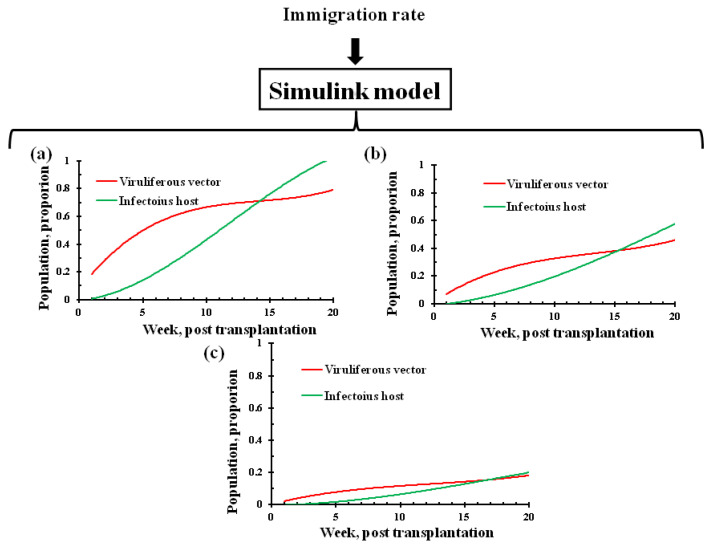
Leaf curl epidemic risk (as infectious host and viruliferous vector population) in relation to migration rate (viruliferous vectors); epidemic level with migration or immigration rate 0.2 (**a**), 0.07–0.1 (**b**), and 0.03–0.05 (**c**) vector/plant/week.

For assessment of the major driver in tripartite interactions, the dynamics of the infectious host and viruliferous whitefly population were predicted at three levels of immigration rate, also known as migration rate parameter (***i_v_***). The migration rate at 0.2 vector/plant/week corresponded with a severe epidemic level within twenty weeks, which was indicated by a high proportion of infectious host and viruliferous whitefly population (Figure 2a). A reduction in the migration rate in the range between 0.07 and 0.1 vector/plant/week showed a remarkable decrease in the level of the infectious host as well as the vector population (Figure 2b). A further reduction in the level of migration rate between 0.03 and 0.05 vector/plant/week appeared to imitate a low level of the epidemic, as both populations remained below 20% (Figure 2c). Finally, the migration rate in the range of 0.0001–0.005 vector/plant/week appeared to be in tune with almost no epidemic, as the infectious population fell drastically.

**Figure 3:**
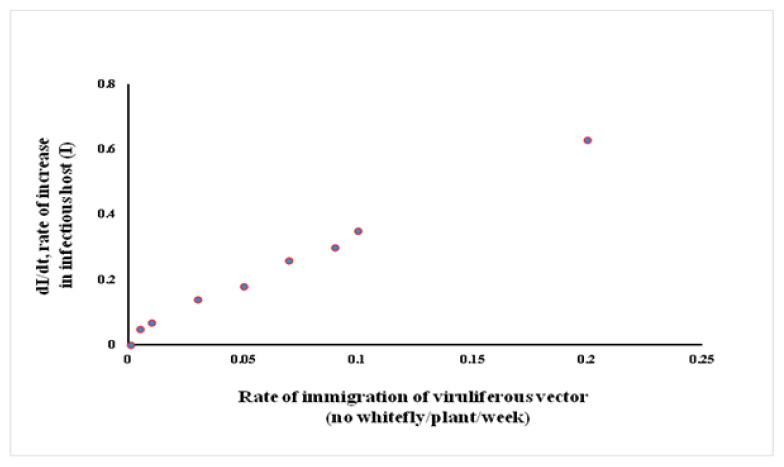
Sensitivity analysis for the migration rate parameter on the infectious host population.

**Figure 4:**
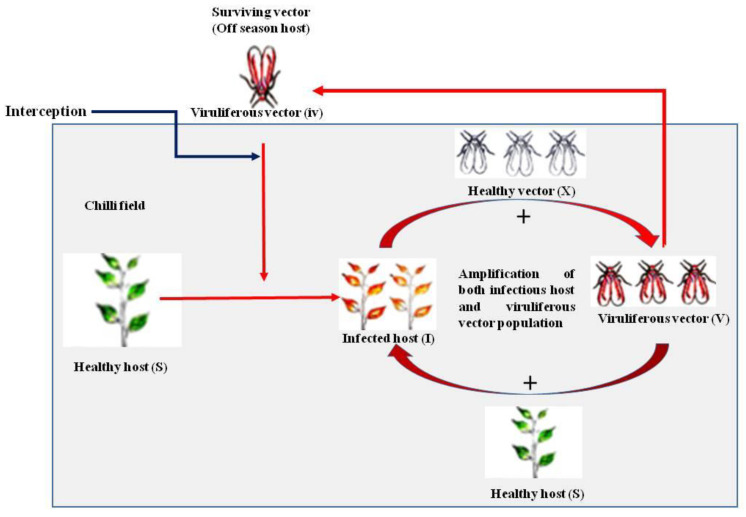
Workable positive feedback loop for the infectious host (*I*) and viruliferous vector (*V*) population incited by the migrant vectors.

The sensitivity analysis indicated that the rate of change in the infectious host population (*I*) was responsive to the change in the number of migrant vectors (Figure 3). A uniform increase in the rate of *I* with the increase in the number of migratory vectors reflected a gradual and simultaneous increase in both the populations, where the initial few viruliferous migrant vectors produced new infections or few infectious hosts, and aviruliferous new generation flies acquired the virus from the newly infected host. Repeated cycles of such virus transfer and acquisition ultimately led to exponential growth for both populations. Therefore, the migrant viruliferous vector appeared to be the driving force in operating the tripartite interactions in an interdependent manner. A positive feedback loop was constructed to explain the phenomenon where few migratory viruliferous vectors produced few infectious hosts, which in turn facilitated more viruliferous vectors; this process continues until the healthy hosts are available in the agroecosystem (Figure 4).

In the population dynamical modelling setup, the migration rate of the viruliferous vector population was noted to be an important link with the leaf curl epidemic. It could be seen that a low rate of immigration, or a low contact rate between the host and vector in the field, is likely to result in a low level of infectious host population. Analogically, a low or no migration rate is expected to result in a low contact rate between the host and viruliferous vector population. A hypothesis that a low or no contact rate at the early stage of transplantation was derived for testing and translation, in order to derive management clues.

### 3.2. Field Assay for Complete Interception and Its Impact on Leaf Curl Epidemic in Chilli during the 2020 and 2021 Season

**Figure 5:**
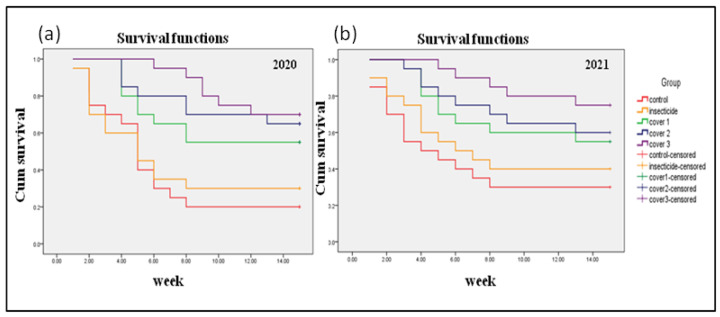
Survival curves (Kaplan–Meier) drawn from the leaf curl observation (under different cover) in the susceptible chilli genotype (HPH-1041) at IARI experimental field in New Delhi during 2020 (**a**) and 2021 (**b**) seasons.

Interception (covering plants) applied for migrant vectors kept plants free of ChiLCV infection for a significantly longer period compared to non-covers. During the 2020 season, the median survival time (0.8) of the plants was 3–4 weeks and nine weeks for the plants that received 15–21 days and 30 days of cover, respectively (Figure 5a). Interception treatment during the 2021 season also ensured similar survival times in 15–21 days as well as in 30 days of cover (Figure 5b). In both seasons, the survival probability curves between different covers and control treatments showed significant differences (*p* < 0.001). A significant log-rank test indicated that an increase in cover periods provided a longer period of protection in terms of survival time (viz., remained free from ChiLCV infection).

The Cox proportional hazard model indicated a significant difference in hazard ratio within different cover periods (Table 1). A significantly higher hazard ratio in the plants was observed with a shorter cover period (15 days). With the increase in the cover period (21–30 days), although the hazard ratio was noted to decrease, the difference was non-significant (*p* < 0.05).

It was evident that complete interception against migrant viruliferous vector population ensured that plants remained healthy for a longer period than those not protected with covers. However, non-significant variation in the hazard ratio between 21- and 30-days’ cover suggested that the time needed for plant cover should be optimized for practical application purposes.

### 3.3. Assessment of Plant Growth Performance under Interception

**Figure 6:**
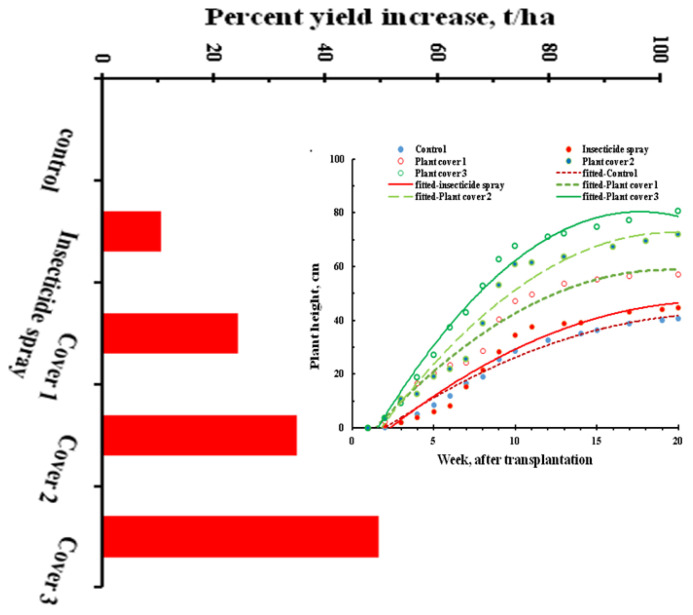
Relative plant growth rate and fresh fruit yield (t/ha) in chilli under interceptions against migrant viruliferous vectors.

Plant growth and yield estimated in terms of relative growth rate (plant height) indicated that plants under cover protection had higher growth rates as compared to unprotected plants (Figure 6). The higher growth rate in those plants also corresponded with a higher fresh fruit yield.

### 3.4. Optimization of Interception Time for Field Implementation

**Figure 7:**
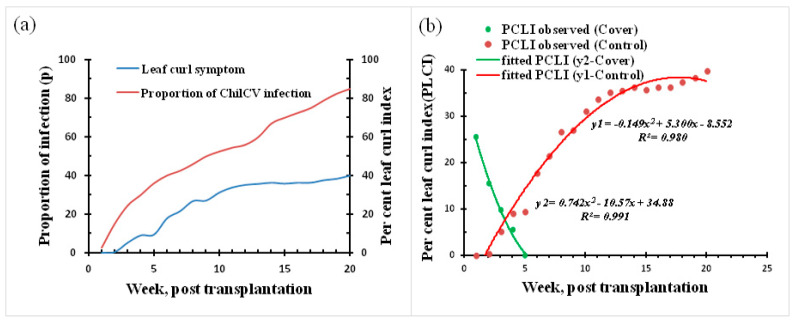
Leaf curl incidence at the IARI experimental field, New Delhi, in 2021; agroecosystem surrounded by susceptible crops, semi-natural perennial habitat, weeds, and borders with cucurbits and legumes. (**a**) Proportion of ChiLCV infection and percent leaf curl index; (**b**) intersect and optimum interception time (days).

Leaf curl incidence in the experimental field (untreated), estimated in terms of leaf curl index (based on symptomatic leaves) and proportion of ChiLCV-infected plants, as detected by PCR, indicated that chilli plants are vulnerable from the time of transplantation. The percent leaf curl index (PLCI) was recorded from the second week post-transplantation, but ChiLCV infection was noted even in the first week of transplantation (Figure 7a). This indicated that chilli plants are prone to whitefly attacks from the first week of transplantation. The infection proportion estimated in the leaf samples indicated a gradual increase until the end of the twentieth week, when around 85% of the samples were infected with ChiLCV. The appearance of typical leaf symptoms within 2–3 weeks suggested that, in addition to whitefly, plants were simultaneously infested by thrips and mites. The percent leaf curl index (PCLI) estimated in the covered plants (14, 21, and 30 days) decreased with an increase in cover time (Figure 7b). The intersect between the polynomials of the percent leaf curl index in the untreated and intercepted field was estimated to be about 3.7 weeks or 26 days.

In a follow-up interception experiment, an optimized cover period (about 26 days) and seed treatment with miticide were observed to be very effective, as plants remained healthy and ChiLCV infection-free until the flowering period. This indicated that the prevention of the migrant vector population from the beginning and the period of interception are very important guides for the formulation of a management strategy.

### 3.5. Prediction of Vector Feeding Rate and Approximation of Contact Rate Dynamics Based on Host Density (Leaf Number and Leaf Area) and Succulence Level

**Figure 8:**
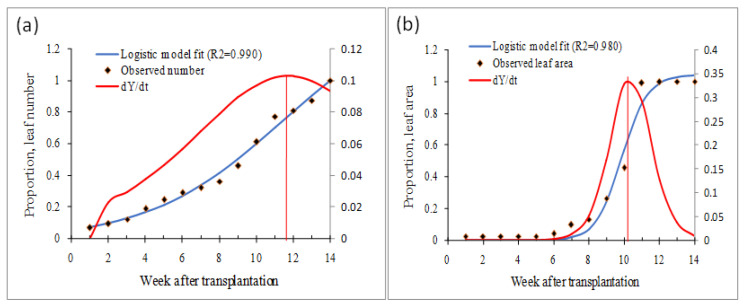
Host density in terms of leaf number (**a**) and area (**b**) in chilli (cv HPH-1041) estimated under controlled conditions.

Host growth dynamics in terms of the number of leaves and area was explained by fitting logistic curve parameter estimates for the leaf number (C = 1.454; a = 0.284; b = 24.232; R^2^ = 0.990) and for the leaf area (C = 1.040; a = 1.386; b = 8.6112E5; R^2^ = 0.980). The number of leaves was noted to increase even after the fourteenth week of transplantation, but the inflection point was reached at around the twelfth week (Figure 8a). The maximum leaf area estimate was noted at around the twelfth week after transplantation, and the inflection point was noted at around the tenth week (Figure 8b). It appears that chilli plants are likely to attain the maximum growth rate both in terms of leaf number and leaf area between 10- and 12-week periods.

**Figure 9:**
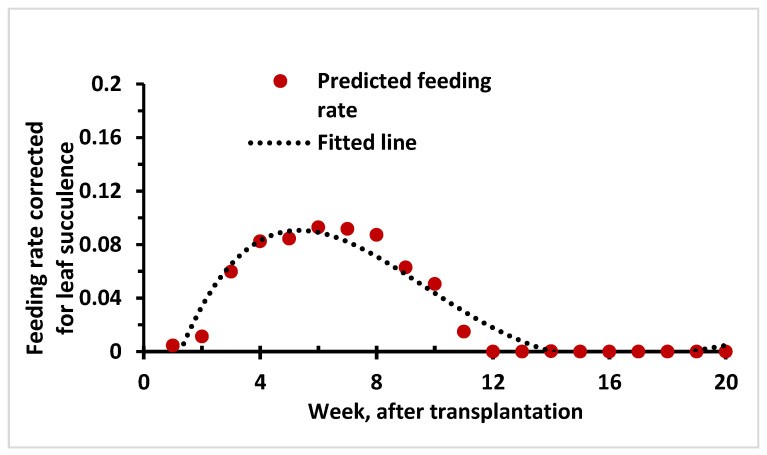
Dynamics of vector predation or feeding rate predicted in chilli based on host density and corrected for host succulence.

The predation rate (or feeding rate) estimated based on the mechanistic model indicated a uniform rise as host density increased over time. However, under natural conditions, predation or feeding rate is subjected to limits as the host growth reaches its saturation. When corrected with succulence level, the feeding rate was shown to reach a peak level at 6 weeks after transplantation and gradually declined afterwards (Figure 9). This indicated that the vector feeding rate, otherwise appropriated as the contact rate, was constrained by host preference due to succulence.

**Figure 10:**
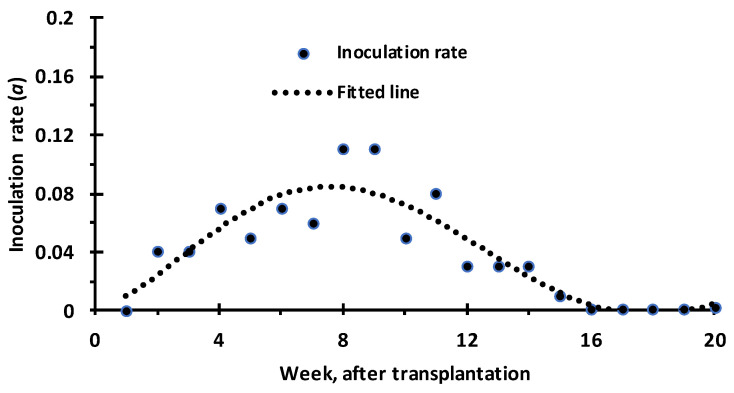
Inoculation rate for ChiLCV in chilli grown under natural field conditions.

The transfer or inoculation rate measured in terms of virus transmission rate from viruliferous vector to healthy host was observed to increase from an initial low rate and reached a peak at 8 weeks after transplantation, followed by a gradual decline (Figure 10). Similarity in the dynamic pattern between the contact and inoculation rate signified the fact that the inoculation rate is dependent on the contact rate. The feeding rate peak in the sixth week and the inoculation rate peak in the eighth week reflected the fact that a gap of 2 weeks’ difference is reasonable considering the virus multiplication to be detected through PCR. Therefore, the reasonable correspondence between the contact rate and the inoculation rate dynamics implied that the host factors, mainly the succulence level, influenced the contact rate.

### 3.6. ChiLCV Transmission Potential in Plants with Different Ages (Leaf-Stage)

**Figure 11:**
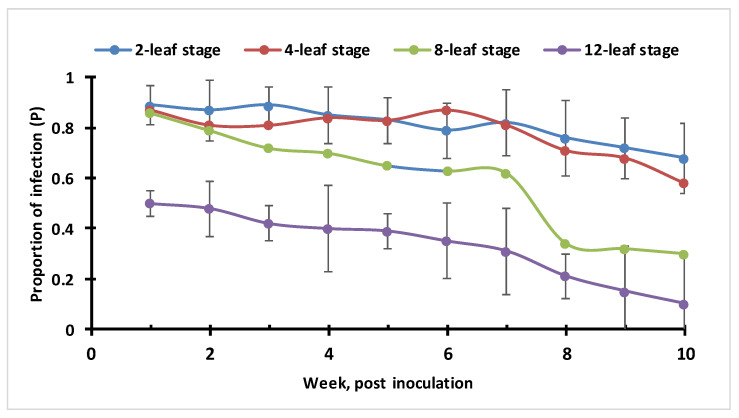
Plant age and ChiLCV transmission potential in susceptible chilli genotype (cv HPH-1041) estimated under semi-controlled conditions.

The ChiLCV transmission potential estimated in different ages of plants (marked by leaf stages) indicated that plant age significantly influenced virus transfers to the host (Figure 11). Plants inoculated at the 2–4 leaf stage showed a significantly higher proportion of infection until the tenth week of post-transplantation compared to plants of the 12-leaf stage (partial F test with *p* < 0.001). The proportion of infection in the eight-leaf stage was between the plants of the 2–4 leaf and 12-leaf stage. This indicated that virus transmission potential in relatively older plants was distinctly lower compared to the younger plants with 2–4 leaf stages. The gradual decrease in virus transmission potential with the increase in age was reflected in feeding preference, or, contact rate between host and vector. Alternatively, it suggested that the protection of plants in an early stage of crop growth is crucial to check the contact rate between the host and vector.

## 4. Discussion

For leaf curl disease in chilli, tripartite interactions between host, vector, and virus, which collectively determine the dynamics of host–vector contact rates, were evaluated and tested to improve management strategy. The interception of the migrant vector population to minimize the contact rate proved to be the cornerstone of the disease management strategy. The time taken for disease appearance with visible symptoms and virus detection indicates that the early plant growth phase is prone to most vectors generally associated with chilli leaf curl syndrome (thrips and mites). Feeding rate prediction and its approximation as a component of contact rate dynamics in chilli were influenced by host growth, particularly succulence level. Further, a decrease in the proportion of infection with plant age also indicated that early plant growth stage is the most vulnerable stage, as supported by the contact rate dynamics. Therefore, an interception from the early period is expected to ensure a no or low level of the epidemic to maintain normal plant growth and fruit yield.

The leaf curl epidemic was revealed to be initiated by the migrant vector population, which is available in the reservoirs of the agroecosystem, and solely contributes to increasing the infectious host as well as the viruliferous vector population in the field. A positive feedback mechanism is in operation where few viruliferous migrant vectors produce few infectious hosts, which in turn, facilitate more viruliferous vectors; this process continues so long as the availability of susceptible host sites is ensured [46,47]. Feeding rate appropriated as contact rate contributes to both virus acquisition and transfers to the host from the time of leaf emergence to the peak level of succulence. An initial low feeding rate is justified by the low host density and increase in rate at a later stage as host density increases. However, the decrease in feeding rate is subsequently modified by the succulence level. The preference of greenhouse whiteflies has been reported to vary with different species of host plants and leaves of different ages, but they mostly prefer the younger leaves [14].

Contact between viruliferous vectors and hosts have been reported to be the crucial step for epidemic development [17,18]. A low or no contact rate is only possible by preventing vector access into the field. Thus, vector interception through plant cover or by any means could ensure epidemic control. The complete avoidance of initial contact between migrant vectors and the healthy host prevents the gradual and simultaneous building of the inter-dependent population. As the gradual build-up of both infectious host and vector starts at the early crop stage, the complete prevention of migrant vectors at the start of chilli planting is expected to deter contact between the two populations, and thus, maintain a low level of virus transmission. The prevention of the infectious vector’s entry from the beginning or complete avoidance of migrant vectors from the crop start may be possible by covering the rows with synthetic plant covers that prevent insects without interfering with plant growth. A precise intervention time that ensures low contact rates resulting in a smaller proportion of infectious hosts is a clue for the evolution of tactics in management strategies [19]. The early plant growth phase is prone to vectors or pests associated with chilli leaf curl disease. The early appearance of symptoms and the detection of associated leaf curl virus suggests that the early growth or succulent stage is a determinant for the visiting of pests or immigration of vectors to a freshly planted field. In agroecosystems, particularly in tropical and semitropical climates where vector reservoirs are maintained throughout the year, immigration is a common phenomenon [48]. Early physiological growth is not only severely affected by viruses, but also due to feeding by whiteflies, thrips, aphids, and mites [49]. In India, eleven viruses are reported to occur naturally on chilli; namely, ChiLCV, cucumber mosaic virus (CMV), pepper venial mottle virus (PVMV), tobacco leaf curl virus (TLCV), potato virus X (PVX), potato virus Y (PVY), tobacco ring spot virus (TRSV), pepper vein banding virus (PVBV), tomato leaf curl New Delhi virus (ToLCNDV), chilli mosaic virus, and capsicum chlorosis virus [1,49,50]. In the current study, although observations were only made for whitefly-transmitted ChiLCV, it may also be applicable to other vectors. Plants kept under complete cover are shown to be devoid of all symptoms except mite injuries. However, remaining completely free of leaf curl symptoms under plant cover and seed treatment (with miticide) indicates that mite injury is a component of leaf curl symptoms. It also appears that covered plants received protection from winged whiteflies and thrips but not from the crawling mites. The occurrence pattern of leaf curl incidence indicates that chilli plants are prone to infection from all pests at the early growth stage, probably due to a feeding preference for host succulence.

Covering plants for around 26 days as an optimum period essentially reduced the initial contact rate and ensured the avoidance of high feeding rate exposure until 6 weeks after transplantation. Location-based trials with plant covers can be standardized, as migrant vector populations may vary depending on the agroecosystems. The reduction in virus transmission with plant age affirms that a short interception period at a critical stage is crucial, as the later stage automatically has less potential for transmission. Further, greater plant growth in symptom-free plants under plant cover strongly suggested that the early protection of chilli plants is crucial to attaining fruit yield. Leaf curl infection reduces the leaf area index, and thus, affects radiation use efficiency and the low accumulation of photosynthates [51]. It thereby impairs the translocation of metabolites, resulting in abnormality at the cell/tissue level, and reduces carbon uptake and soluble assimilates, ultimately impairing crop growth [51,52].

To summarise, the time of host–vector contact is a crucial meeting point where multiple influencing factors interact and give rise to leaf curl infection, knowledge of which has been identified as critically lacking. The migrant vectors, as the primary drivers of the ChiLCV epidemic, and the contact rate, as the function of host density and succulence, have been proved and translated into rules for the evaluation of a management strategy. In the future, location-based trials with plant covers can be standardized, as migrant vector populations may vary depending on the agroecosystems. Moreover, future improvements in the epidemiologically focused management of ChiLCV should also consider other factors affecting contact and virus acquisition rate (i.e., bite exposure rate, as per Thongsripong et al., 2021) [20], the effect of plant canopies and physiology, the varietal preference of whiteflies to chilli, and environmental factors affecting vector feeding behaviour as well as reproduction. Such studies will further narrow the view of researchers to assess management choices and determine the best option to contain the epidemic spread of the disease.

## Figures and Tables

**Table 1:** Cox models for different periods of plant cover (interception) in relation to leaf curl infection in susceptible chilli genotype (HPH-1041) observed at IARI experimental field, New Delhi, in 2020 and 2021.

Explanatory Variable (Groups of Treatments)	2020				2021			
	Hazard Ratio	Standard Error	95% CI	*p*-Value	Hazard Ratio	Standard Error	95% CI	*p*-Value
Control (without any treatment)								
Insecticide spray (imidacloprid 0.01%)	5.60	0.52	2.02–15.52	0.001	5.02	0.52	1.80–13.99	0.002
Plant cover (interception for 14 days)	3.72	0.53	1.31–10.60	0.014	3.58	0.53	1.26–10.19	0.017
Plant cover (interception for 21 days)	2.23	0.55	0.74–6.67	0.15	2.12	0.55	0.71–6.32	0.178
Plant cover (interception for 30 days)	1.55	0.58	0.49–4.91	0.44	1.78	0.57	0.58–5.44	0.312

## Data Availability

The data that support the findings of this study are openly available: https://github.com/9873018656 (accessed on 30 March 2022).

## Supplementary Materials

The following supporting information can be downloaded at: https://www.mdpi.com/article/10.3390/v15040854/s1, Table S1: Model parameters and variables used for leaf curl epidemic analysis in chili; Table S2: Sampling procedure for estimation of proportion of infection in leaf and whitefly population through PCR detection of ChiLCV.
