# Supplementary material for: Leaf Curl Epidemic Risk in Chilli as a Consequence of Vector Migration Rate and Contact Rate Dynamics: A Critical Guide to Management
Source: Viruses. 2023 Mar 27;15(4):854. doi: 10.3390/v15040854 (PMC10144731; doi:10.3390/v15040854)
Supplement: Supplementary file 1 [file viruses-15-00854-s001.zip › viruses-2245937-supplementary.pdf]

Table S1. Model parameters and variables used for leaf curl epidemic analysis in chili.

Symbol	Name	Estimated range/adjusted
a	Transmission rate	0.057 - 0.10 plant $vector^{-1}$ day^{-1}
$\boldsymbol{K_x}$	Michaelis-Menten for rate of change in healthy vector (*X*)	2.8 healthy vector $week^{-1}$
b	Acquisition rate	0.1115 vector $plant^{-1}$ h^{-1}
$\boldsymbol{i_v}$	Immigration rate for viruliferous vector	0.34 vector $week^{-1}$
$\boldsymbol{e_v}$	Emigration rate for viruliferous vector	0.005 vector $week^{-1}$
$\boldsymbol{i_x}$	Immigration rate for healthy vector	0.05 vector $week^{-1}$
$\boldsymbol{e_x}$		0.005 vector $week^{-1}$
u	Death rate for healthy as well as viruliferous vector	0.34 vector $week^{-1}$
c	Birth rate for healthy as well as viruliferous vector	0.05 vector $week^{-1}$
S	Healthy plant (1-*I*)	0-1.0
I	Infectious plant (PCR- positive)	0-0.99
V	Viruliferous vector (PCR- positive)	0.65-0.97
S*	Healthy plant (1-*I**) at equilibrium	0-1.0
X*	Healthy vector (1-*V*)	0-1.0
V*	Viruliferous vector population at equilibrium	0-1.0
I*	Infectious population at equilibrium	0-1.0

Table S2. Sampling procedure for estimation of proportion of infection in leaf and whitefly population through PCR detection of ChiLCV.

<table>
<tr><td rowspan="2">Step</td><td colspan="2">Composite sampling and PCR detection for ChiLCV</td></tr>
<tr><td>Leaf</td><td>Whitefly (Wf)</td></tr>
<tr><td>1</td><td>200 leaves collected from the individual plant</td><td>100 Wf collected from the individual plant</td></tr>
<tr><td rowspan="5">2</td><td>Divided into two groups of 125 and 75 leaves.
• First, 125 leaves again divided into 5 groups consists of 25 leaves.
• Remaining 75 leaves divided into 3 groups consists of 25 leaves in each group.</td><td>Divided into two group of 70 and 30 Wf.
• First, 70 Wf divided into 7 groups consists of 10 Wf.
• Remaining 30 Wf divided into 3 groups consists of 10 flies in each group.</td></tr>
<tr><td colspan="2">PCR detection using degenerate primers pairs</td></tr>
<tr><td colspan="2">If not all PCR-positive (in first 5 groups) then follow PCR detection for the remaining 3 group</td></tr>
<tr><td colspan="2">Calculate $P^* = 1-((n-X)/n)^{1/m}$; n= number of groups; m= number of leaves or whiteflies in each group; X= number of groups tested PCR- positive.</td></tr>
<tr><td>n=(5+3)=8
m=25</td><td>n=7+3=10
m=10</td></tr>
<tr><td rowspan="4">3</td><td>If all 5 groups PCR-positive in step 2 then remaining 75 leaves divided in to two groups of 50 and 25 leaves and PCR detection.</td><td>If all 5 groups PCR-positive in step 3 then remaining 30 WF divided in to two groups of 20 and 10 WF and PCR detection.</td></tr>
<tr><td>Group of 50 divided into 10 groups each group having 5 samples</td><td>Group of 20 divided into 5 groups each group having 4 samples</td></tr>
<tr><td colspan="2">PCR detection using degenerate primers pairs</td></tr>
<tr><td>If not all PCR-positive (in 10 groups) then calculate P* with n=10 and m=5</td><td>If not all PCR-positive (in 5 groups) then calculate P* with n=5 and m=4</td></tr>
<tr><td rowspan="2">4</td><td>If all 10 groups PCR-positive in step 3 then for the remaining 25 leaves PCR detection for each leaf.</td><td>If all 5 groups PCR-positive in step 3 then for remaining 10 WF, PCR detection with single fly.</td></tr>
<tr><td>Calculate P* as simple proportion out of 25 leaves</td><td>Calculate P* as simple proportion out of 10 flies</td></tr>
<tr><td colspan="3">PCR METHOD:
For ChiLCV detection, genomic DNA was isolated from the leaf and whitefly samples (CTAB method). PCR was performed in 25 µl reaction mixture containing 20-30 ng genomic DNA, 2.5µl of 10X PCR buffer (Thermo Scientific), 1 µl of each forward (GEM-F) and reverse (GEM-R) primers (Eurofins), 260 µM of dNTP (Thermo Scientific) and 2U of DreamTaq polymerase (Thermo Scientific). Degenerate pair primers (GEM-F: 5’-ATRRTHTGGATGGAYGARAACAT-3’;GEM-R:5’AAATCCCCTNTATTTCAAARAT-3’) was used to amplify approximately 760 bp sequence partially overlapping the putative AV1, AC3 and AC2 genes (Roy et al., 2015) following amplification steps: 94ºC for 2 m, 35 cycles of 94ºC for 30 s, 48ºC for 45 s and 72ºC for 30s; 72ºC for 10 m (Thermocycler, Hi-Media). PCR-positive of a sample was confirmed by the presence of specific (760 bp) band in agarose gel (2%).</td></tr>
</table>
